# Effect of virtual reality exercises on balance and fall in elderly people with fall risk: a randomized controlled trial

**DOI:** 10.1186/s12877-021-02462-w

**Published:** 2021-09-25

**Authors:** Noorolla Zahedian-Nasab, Azita Jaberi, Fatemeh Shirazi, Somayyeh Kavousipor

**Affiliations:** 1 Student Resarch Committe, School of Nursing and Midwifery, Shiraz University of Medical Sciences, Shiraz, Iran; 2 Community Based Psychiatric Care Research Center, School of Nursing and Midwifery, Shiraz University of Medical Sciences, Shiraz, Iran; 3 Community Based Psychiatric Care Research Center, School of Nursing and Midwifery, Shiraz University of Medical Sciences, Shiraz, Iran; 4 Occupational Therapy Department, School of Rehabilitation Sciences, Rehabilitation Sciences Research Center, Shiraz University of Medical Sciences, Shiraz, Iran

**Keywords:** Aged, Exercise, Virtual reality, Postural balance, Fall, Nursing homes, Xbox

## Abstract

**Background:**

Deficient balance and fear of falling in elderly people can lead to disturbed daily activities, falling, and finally reduced quality of life. Therefore, evaluation of low-risk methods that might partially improve balance in this group of people is of utmost importance. The present study aimed to investigate the impact of Virtual Reality (VR) exercises based on Xbox Kinect on balance and fear of falling among elderly people.

**Methods:**

This clinical trial was performed on 60 elderly individuals living in nursing homes divided into two groups of control and Xbox. The participants in the intervention group received VR exercises based on Xbox Kinect in form of two 30–45-min sessions held on a weekly basis for 6 weeks. The individuals in the control group, on the other hand, received routine exercises of the nursing homes. The research tools used in this study included a demographic questionnaire, the Berg Balance Scale (BBS), the Timed Up and Go (TUG) test, and the Falling Efficacy Scale (FES).

**Results:**

The findings of the current study demonstrated that the scores of BBS and TUG test as the indices of balance among elderly people improved significantly in the Xbox group after the intervention (*p* < 0.001 for both BBS and TUG test). Moreover, the score of fear of falling diminished significantly in the intervention group compared to the control group (p < 0.001).

**Conclusion:**

According to the results of the present investigation, 6 weeks of VR balance exercises could enhance balance and fear of falling among elderly people living in nursing homes.

**Trial registration:**

Code:IRCT20190727044347N1, Date: 17-8-2019.

## Background

In the recent decades, advances have been made in medicine, technology, and public health and the knowledge of people about health, nutrition, and education has improved. As a result, life expectancy has increased throughout the world and the world population has moved towards getting old [1]. Elevated life expectancy has in turn led to a diminished mortality rate, an augmented lifetime, and problems concerning the quality of life among elderly people [2]. One of the problems associated with a longer lifetime is falling. Falling is among the reasons for injury and death in elderly individuals, as one out of every three people over 65 years old and one out of every two individuals over 80 years old experience falling during a year [3–5]. According to the literature, falling among elderly people imposes heavy expenses both directly (medical) and indirectly (non-medical) on people and the society [6, 7]. Fracture, performance limitations, traumatic injuries to the brain, disability, extra expenses, and mortality are among the most important sequels of falling [8].

Falling in elderly individuals is affected by diverse factors, some of which can be balanced, while some cannot. Fear of falling and impaired balance are known as the adjustable risk factors of falling in old people [9]. Fear of falling is one of the common problems in elderly people and is more frequently observed in individuals with an experience of falling, impaired balance, low social activities, depression, and weak autonomy [10, 11]. Studies have indicated that about half of elderly individuals have experienced falling at least once [12, 13]. Fear of falling results in limitations in physical activities, which might be followed by falling. Consequently, the quality of life decreases and a low-mobility lifestyle occurs [14]. In addition to the fear of falling, impaired balance is one of the most prevalent reasons for falling amongst elderly people [15]. Disturbed balance results in the fear of falling in elderly people, which can lead to impaired daily activities and diminished quality of life [3, 5]. Disturbed balance, which is another reason for falling in elderly people [15], has been defined as the disability for maintaining balance and alterations in body consistency at the center [16].

There are various methods for maintaining or enhancing balance and some of the common therapeutic techniques include tango dance [17, 18], yoga [19, 20], tai chi [21–23], and video games. Considering the tendency of modern society towards computer and video games played by smart computers [24–28], video games have been recently noted as a substitute for the rehabilitation of disabled people. Some of the benefits of using smart computers for improving balance entail the possibility of easy application at home, lower costs compared to other therapeutic methods, and better acceptance of the intervention by individuals due to being interesting [25]. Xbox Kinect is a smart computer that can simulate balance exercises. This computer can recognize people’s movements by a camera and infrared motion sensors and helps people perform movements correctly in case they are wrong. Moreover, this smart computer allows people to move freely and have diverse positions [27]. The Xbox Kinect can be used for rehabilitation purposes as well as for exercising (also termed “exergames”) [29].

Considering the elevated population of elderly people in modern societies and the high prevalence of falling in this group, exercises that can promote the physical performance of these people and decrease the risk of falling have been taken into consideration by health caretakers. Therefore, it is highly important to evaluate low-risk methods, which can partially improve the balance of these individuals and finally enhance their quality of life [30]. Some studies in this regard have demonstrated that video exercises might improve physical performance and balance [25, 31], eventually leading to a reduction in the fear of falling [12, 15, 32]. For instance, Bieryla (2016) conducted a pilot study on healthy elderly individuals from living communities and an intervention group trained with the Kinect for Xbox 360 for 3 weeks. The results showed significant changes in Berg Balance Scale (BBS) and Fullerton Advanced Balance (FAB) [31]. Another systematic review also showed the positive effects of Kinect systems on rehabilitation for elderly people suffering from stroke and falling risk [33]. However, most of these studies were pilot investigations with small sample sizes and short intervention durations. Moreover, studies conducted in Iran have assessed the effect of video games on elderly people with stroke [34]. Elderly people living in nursing homes, on the other hand, may suffer from balance disorders and fear of falling for reasons other than stroke. Therefore, studying the effect of these methods among elderly people with various disorders could provide a more appropriate conclusion about this type of exercise for researchers. According to Neil et al., there are significant differences in the physical interaction and therapeutic emphasis of diverse games [35].

Nurses have an important role in ensuring the safety of their clients and face many challenges in this regard [35]. Given that lack of balances and falls are threatening factors for the safety of the elderly, it is necessary for nurses to investigate the impact of various interventions on reducing these threats. Considering the importance of this issue and the lack of investigations in this regard, the present study aims to evaluate the impact of Virtual Reality (VR) exercises on balance and fear of falling among the elderly people living in the nursing homes.

## Methods

### Study design and setting

This clinical trial with a pretest-posttest design was performed in the nursing homes of Shiraz, Iran in 2019.

### Participants

The participants of the current study were males and females aged over 60 years living in nursing homes. First, all interested elderly people were invited to participate in the research. Afterwards, the interested individuals were tested using the Timed Up and Go (TUG) test, and 60 people with TUG scores of 14–20 were selected [36] based on the inclusion and exclusion criteria. The initial TUG test scores of the participants were recorded for comparison to post-intervention scores.

The inclusion criteria of the study entailed the ability to walk with or without assisting tools and the permission of the doctor at the nursing home. The exclusion criteria included a history of acute and chronic physical, cognitive, and mental diseases that might hinder exercising, participation in other exercises similar to the intervention, having problems leading to problematic exercises, unmodified hearing and seeing problems, and balance disorders due to the problems of the vestibular system and cerebellum diagnosed by a doctor.

### Ethical consideration

The present study was approved by theEthics Committee of Shiraz University of Medical Sciences (IR.SUMS.REC.1398.573). It was registered in Iranian Registry of Clinical Trials (IRCT) with registration number of IRCT20190727044347N1 on 2019-08-17. All necessary permissions for conducting the research were obtained from the relevant administrators and all methods were performed in accordance with the relevant guidelines and regulations. Furthermore, a session was held after the selection of participants for explaining the study objectives and procedures. Written informed consent forms were also taken from all participants.

### Sample size

According to the research by Park et al. (2017), using the equation for the difference of BBS means (mean of 50 ± 6.27 for the intervention group and 44.7 ± 7.47 for the control group), and considering type 1 error of 0.05 and power of 80%, the sample size was calculated as 27 for each group. Considering dropout, 30 participants were allocated to each group, making a total sample size of 60 [37].

### Randomization

The participants were divided into an intervention group (i.e., exercise by Xbox Kinect) and a control group (i.e., routine programs of the nursing homes) through random allocation using double randomized permutation block with foursome blocks. The random list was generated by a statistician using the “Random Allocation” software, and 15 blocks were selected for forming the two study groups. The list was then provided to the researchers, and one of them performed the randomization accordingly.

### Interventions

The participants were asked to fill out the demographic questionnaire, Falling Efficacy Scale (FES), and TUG test. It should be noted that the TUG test results were collected at the stage of sample selection. Afterwards, the balance of all participants was measured and recorded. Next, the intervention group received simulated balance exercises in the form of two 30–60-min sessions on a weekly basis for 6 weeks. For simulated balance exercises, Xbox Kinect was applied, which is a game console simulating balance exercise in the game environment and allows a person to move freely and have diverse positions. This computer recognizes and executes the movements of people via a camera and infrared motion sensors. This smart computer contains a variety of games. In this study, suitable games for improving the balance of elderly people were selected in a meeting with the professors of the Rehabilitation Department. The selected games (Kinect Sports 1 and 2) included penalty, goalkeeping, ski, and darts, and each session covered a different aspect of enhancing balance. All selected exercises required the application of upper and lower organs while standing (Table 1).

**Table 1:** Description of each game in the Xbox Kinect sports pack

Ski	Weight shifting to the right and left or up and down is trained. The screen demonstrates a virtual slope and players should follow the slope without crashing into the barriers.	– Active movement of the lower extremity (flexion, abduction, and external-internal rotation of hip; flexion and extension of knees; dorsiflexion and plantar flexion of ankles) – Trunk rotation – Training weight-shifting and weight-bearing – Training balance
Penalty and goalkeeper	The usage of lower extremities, head, neck, and trunk while standing and kicking a ball in a virtual soccer field is practiced.	– Active movement of the lower extremities (flexion, abduction, and external-internal rotation of hip; flexion and extension of knees; dorsiflexion and plantar flexion of ankles) – Training weight-shifting and weight-bearing – Training balance
Darts	Players throw small missiles known as darts at a circle-shaped dartboard. Three darts are utilized per visit at the board to reduce 501 to 0.	The upper limb has unilateral movements along with static standing.

In order to perform simulated exercises in each session, first, the position of the individual was set at 1.5–2 m from the computer and the exercise was explained to the person. Prior to the exercises, permission was taken from the doctor of the nursing home. Exercises were stopped in case of fatigue, pain, or dyspnea. During the game, the participants were encouraged by the researcher. It is worth mentioning that special mats were used in order to prevent injury to the elderly people. The exercises were continued for 30–60 min each session and all participants in the intervention group played all specified games for 6 weeks.

In the control group, the elderly individuals received the routine programs of nursing homes, including jogging in the nursing home, table tennis, and some artistic activities, for 6 weeks. After 6 weeks of intervention, balance, physical performance, and fear of falling were assessed in the participants of both control and intervention groups and were compared to the data recorded previously.

### Outcome measures and follow-up

Data collection tools in the present study included a demographic questionnaire, FES, Berg Balance Scale (BBS), and the TUG test. The demographic questionnaire encompassed age, gender, previous employment status, education level, marital status, duration of stay at the nursing home (direct question from elderly people), and a record of the number of fallings during the previous year.

Fear of falling was evaluated using FES, which addressed the rate of fear of falling during different daily activities. The answers ranged from “I am worried a little” [1] to “I am worried very much” [4]. Thus, the scores of the survey could range from 16 to 64, with higher scores showing a greater fear of falling. The validity and reliability of the English and Persian versions of this tool have been evaluated and confirmed in the previous studies.

The participants’ balance was assessed utilizing BBS, which is a clinical test for evaluating people’s static and dynamic balance. This survey contained 14 items responded based on a five-point Likert scale, in which four indicated the best execution and zero demonstrated the worst execution. The total score of the scale could range from zero to 56 and was obtained by summing up all 14 items. The psychometric characteristics of this instrument were investigated and confirmed in the previous studies in Iran [38].

The participants’ balance in walking was evaluated by applying the TUG test that examined distinct aspects of static and dynamic balance during daily activities. This test included three stages of standing up, walking three meters, turning around, and returning. The time of the test, as the dependent variable, was measured by a timer. When the participants announced that they were ready, the timer was started and when they returned and their backs touched the chair, the timer was stopped. The validity and reliability of this tool have been assessed and confirmed in various studies [39, 40].

### Statistical analysis

The normality of the data was assessed using the Kolmogorov-Smirnov test, and the results indicated that all demographic data and other study variables had normal distribution. Descriptive statistics were used for the demographic data and the variables were reported as mean, standard deviation, frequency, and percentage. Independent t-test and chi-square test were applied for comparing the two groups regarding the demographic variables. Moreover, the pre- and post-intervention scores were compared in the two groups via paired t-test. To compare the difference between the scores of the two groups, independent t-test and Analysis of Covariance (ANCOVA) were utilized.

## Results

**Fig. 1:**
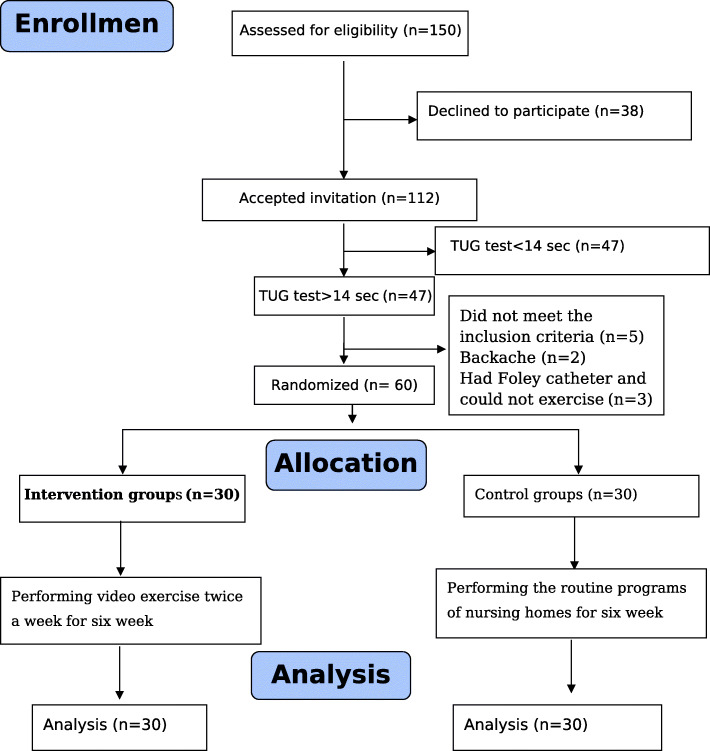
CONSORT flow diagram of the clinical trial

A total of 150 elderly people were candidate for participating in the current study, among whom 112 people expressed a tendency for participation. The TUG test was conducted for these individuals, 65 of whom had a test time of over 14 s and could be enrolled into the study. Afterwards, these people were evaluated in terms of other inclusion criteria and finally, 60 (16 females and 44 males) were selected and divided into a control and an intervention group through random allocation (Fig. 1). After 6 weeks of intervention, balance, physical performance, and fear of falling were assessed in the participants of both control and intervention groups and were compared to the data recorded previously.

The mean age of the participants was 70.83 ± 7.79 years and most of them were male (73.3%) and single (45%). The demographic characteristics of the participants have been presented in Table 2. Accordingly, no significant difference was found between the two groups in terms of age, gender, marital status, education level, occupation, duration of stay at the nursing home, and number of falls before the intervention (*p* > 0.05).

**Table 2:** Demographic data of the participants

Variable	Intervention (***n*** = 30)	Control (***n*** = 30)	***P***-value
Age (year) (Mean ± SD)	69.67 ± 7.725	72 ± 7.808	0.249
Duration of stay at nursing homes (month) (Mean ± SD)	22.34 ± 20.43	16.97 ± 18.34	0.514
Number of falls during the previous year (Mean ± SD)	2.10 ± 1.49	2 ± 1.46	0.794
Gender			1
Male	22 (73.3)	22 (73.3)	
Female	8 (26.7)	8 (26.7)	
Marital status n (%)			0.209
Single	12 (40)	15 (50)	
Married	11 (36.7)	4 (13.3)	
Widowed	6 (20)	9 (30)	
Divorced	1 (3.3)	2 (6.7)	
Education level n (%)			0.342
Illiterate	9 (30)	10 (33.3)	
Primary school	12 (40)	7 (23.3)	
Junior high school and high school	5 (16.7)	11 (36.7)	
Academic degree	4 (13.3)	2 (6.7)	
Previous occupation n (%)			0.658
Employee	6 (20)	10 (33.3)	
Worker	7 (23.3)	5 (16.7)	
Freelance	11 (36.6)	10 (33.3)	
Homemaker	6 (20)	5 (16.7)	

Comparison of the study variables, including pre- and post-test balance, TUG test, and FES, has been summarized in Table 3. Accordingly, the mean score of balance significantly augmented in the intervention group post intervention (*p* < 0.001), while this elevation was not observed in the control group (*p* = 0.687). In addition, the mean balance changes were significantly different between the control and intervention groups (*p* < 0.001). Considering the results showing that the two groups were not similar in this regard before the intervention, ANCOVA was used. The impact of the intervention on balance in the intervention group was significant even with the pre-test effect control (*p* < 0.001).

**Table 3:** Comparison of the two groups regarding the mean scores of BBS, TUG test, and FES

Variable	Groups	Baseline (Mean ± SD)	Six weeks (Mean ± SD)	Change (Mean ± SD)	***P***-value^**a**^ (within-group)
**BBS**	Intervention	36.9 ± 8.6	40.4 ± 7.7	3.4 ± 2.76	< 0.001
	Control	31.7 ± 9	31.9 ± 7.8	0.2 ± 2.6	0.68
*P*-value (between-group)		0.026 ^b^	< 0.001 ^c^	< 0.001 ^c^	–
**Balance state (TUG test)**	Intervention	15.3 ± 1.9	13.0 ± 2.6	−2.3 ± 1.6	< 0.001
	Control	16.6 ± 2.7	16.7 ± 2.6	0.06 ± 2.5	0.88
*P*-value (between groups)		0.04^b^	< 0.001 ^c^	< 0.001 ^c^	–
**FES**	Intervention	37.6 ± 11.2	33.5 ± 9.9	−4.0 ± 4.4	< 0.001
	Control	41.4 ± 11.6	42.3 ± 9.6	0.8 ± 3.6	0.217
*P*-value^b^(between groups)		0.195	0.001	< 0.001	

*BBS* Berg Balance Scale, *TUG* Timed Up and Go, *FES* Falling Efficacy Scale

^a^ Paired t-test for within-group comparisons; ^b^Independent t-test; ^c^ ANCOVA for between-group comparisons

The study findings revealed that the mean of the post-intervention TUG test reduced by about 2.33 s in the intervention group (*p* < 0.001), while the two groups were not significantly different (*p* = 0.88). Considering the difference between the two groups at the beginning, ANCOVA was utilized. The influence of the intervention on this factor was found to be significant with the pre-test effect control (*p* < 0.001).

The results indicated that the mean score of FES was similar in the two groups prior to the intervention. However, the mean score of this factor decreased significantly following the exercises (*p* < 0.001), but did not change in the control group (*p* = 0.217).

## Discussion

According to the results of the present investigation, simulated balance exercises could lead to the enhancement of balance, TUG test, and FES among the elderly people with impaired balance. The findings revealed that virtual exercises resulted in the improvement of the mean score of balance in the participants, as BBS was enhanced. Other studies also showed that utilization of Xbox smart computer could promote balance and reduce the risk of falling in old people [24], video games could improve motor function in patients with cerebral infarction [37] and enhance dynamic balance in children with ataxia [41]. A meta-analysis on elderly people indicated that sport interventions could diminish the fear of falling at a low to moderate level [15].

However, Ki-hun-cho et al. demonstrated that although video exercises enhanced dynamic balance, no significant impact was noted on the static balance of the individuals under investigation [42]. Virtual games are mentally and physically challenging for people and the direction, rate, and speed of movements alter constantly during the games. As a result, rapid mental processing and body balance control along with fast changes are required [43].

Most of the games used in the current study needed the active movements of the hip joint, knees, ankles, and different muscles. A higher rate of using these joints and muscles along with more efforts of elderly people to concentrate on games could enhance balance among the participants. Moreover, receiving real-time and individual feedbacks from the system based on balance status resulted in the participants’ more focus on their balance and further attempts to enhance their balance in future games. It is worth mentioning that exercise might result in diminished fallings and fear through improving power, stepping, balance, and mood [12]. Furthermore, receiving visual feedbacks in simulated exercises could lead to the elevation of the participants’ awareness of their balance control and enhancement of their self-efficacy [44].

In the present study, the TUG test that consisted of three stages and was another index of balance was over 14 s in both groups at the beginning, demonstrating balance disorder in both groups. However, the TUG test decreased significantly in the intervention group after the intervention. These results were in line with those of the research by Htut et al. [44] concerning the effect of virtual exercises on the physical, cognitive, and functional status of elderly people. These authors reported that the TUG test was significantly reduced in the intervention group compared to the control group. Moreover, Park et al. [37] indicated that simulated exercises by smart Xbox Kinect could improve the TUG test among people with hemiplegia. Yang et al. [45] also stated that VR exercises had impacts similar to those of real exercises, and might enhance balance. They concluded that virtual exercises could be a suitable substitute for real training. However, Bieryla et al. [31] showed that video games promoted balance among elderly people based on BBS and Fullerton Advanced Balance Scale, while TUG and functional tests did not change. According to the results, virtual exercises might improve walking performance and tolerance through increasing people’s activity and engagement in games [37].

The current study results demonstrated that simulated exercises significantly diminished the fear of falling amongst elderly participants in addition to improving their balance. The latter finding was consistent with that of the study by Levy et al. and Singh et al. who showed that virtual exercises enhanced the fear of falling among elderly people [43, 46]. However, in the Kwok et al. study, these exercises did not diminish the fear of falling in elderly individuals in 12 weeks, but reduced the fear after 24 weeks [47]. Nonetheless, Rodriguez et al. assessed the influence of dance by Xbox Kinect on the fear of falling in elderly people and revealed that dancing with video games did not affect the fear of falling among these people [48]. In justifying these inconsistent results, Hornyak et al. believed that the fear of falling in old people depended on their physical performance, and older age resulted in increased fear of falling due to declined physical performance [49].

Participating or memory of participating in challenging activities, such as virtual sports, might promote balance confidence and decrease the fear of falling [48]. No improvement in the balance and fear of falling in the control group in the present study highlighted the necessity for elderly people to be physically active. The lack of sports activities in these people might cause impaired balance and augmented fear of falling, leading to a higher rate of falling [30].

Overall, the results of the present study showed that video exercises could improve elderly people’s balance and fear of falling. The impact of video exercises, as an interesting tool [14, 50], could provide a guide for the treatment team for the enhancement of these variables. As a result, the most is made of small spaces in institutes, which do not have sufficient space for other exercises or when elderly individuals are not interested in other sports.

### Strong points and limitations

The tendency of elderly individuals for participating in the exercises in the current study was among the strong points of the research. Moreover, the participants stated that these exercises resulted in competition and happiness. Nevertheless, a limitation for this investigation was a disturbance in the sensor of the Xbox Kinect in some situations, which caused the researcher to be mistaken by a participant in some situations and made him keep the distance to solve this problem. The difficulty of exercises for some participants at the beginning was another study limitation. Additionally, Kinect systems are not suitable for patients with severe disabilities [29]. Considering the fact that there were no severely disabled patients in the present study, the effect of the VR on theses patients’ outcomes could not be measured. One other study limitation was the lack of traditional exercises, such as Otago and tango dance, for comparison of their efficiency to that of the exercises used in the research. This might be taken into consideration in future studies. Furthermore, some researchers have pointed out some limitations of this type of intervention, including the fixed location of the sensor with a range of capture of roughly 10 meters, difficulty in fine movements capture, and indirect fall-risk assessment [29]. Given that researchers in most studies have focused on improving the mobility of patients and advantages of this intervention, it is necessary to consider more realistic and specific results. Moreover, rehabilitation objectives, including fine motor skills, cannot be evaluated by Kinect alone, and these results should be accompanied by those of other evaluation methods. Of course, games were not used as a rehabilitation strategy in the present study. However, if they are used for rehabilitation, other evaluation methods, such as elderly people’s opinions, should be used.

## Conclusion

The findings of the present study demonstrated that simulated balance exercises could lead to the enhancement of balance, TUG test, and FES in elderly individuals with balance disorder. Considering the efficacy of virtual balance exercises in improving the balance of elderly people and some positive features of such exercises, they can be utilized in nursing homes. Some of these positive points include easiness to use and lower costs compared to some therapeutic methods. Nonetheless, further investigations for evaluating diverse virtual exercises and their impacts on different physical and psychological aspects compared to traditional exercises are warranted.

## Data Availability

The datasets used and/or analyzed during the current study are available from the corresponding author on reasonable request.

## Abbreviations

ANCOVA: Analysis of Covariance
BBS: Berg Balance Scale
FES: Falling Efficacy Scale
TUG: Timed Up and Go
VR: Virtual Reality
